# Supplementary material for: Population genomics of Puccinia graminis f.sp. tritici highlights the role of admixture in the origin of virulent wheat rust races
Source: Nat Commun. 2022 Oct 21;13:6287. doi: 10.1038/s41467-022-34050-w (PMC9587050; doi:10.1038/s41467-022-34050-w)
Supplement: Supplementary file 1 — Supplementary Information [file 41467_2022_34050_MOESM1_ESM.pdf]

Supplementary Material

Guo et al. Population genomics of *Puccinia graminis* f.sp. *tritici* highlights the role of admixture in the origin of virulent wheat rust races

Corresponding authors: Les Szabo, e-mail: lszabo@umn.edu; Eduard Akhunov, email: eakhunov@ksu.edu

Supplementary Figures

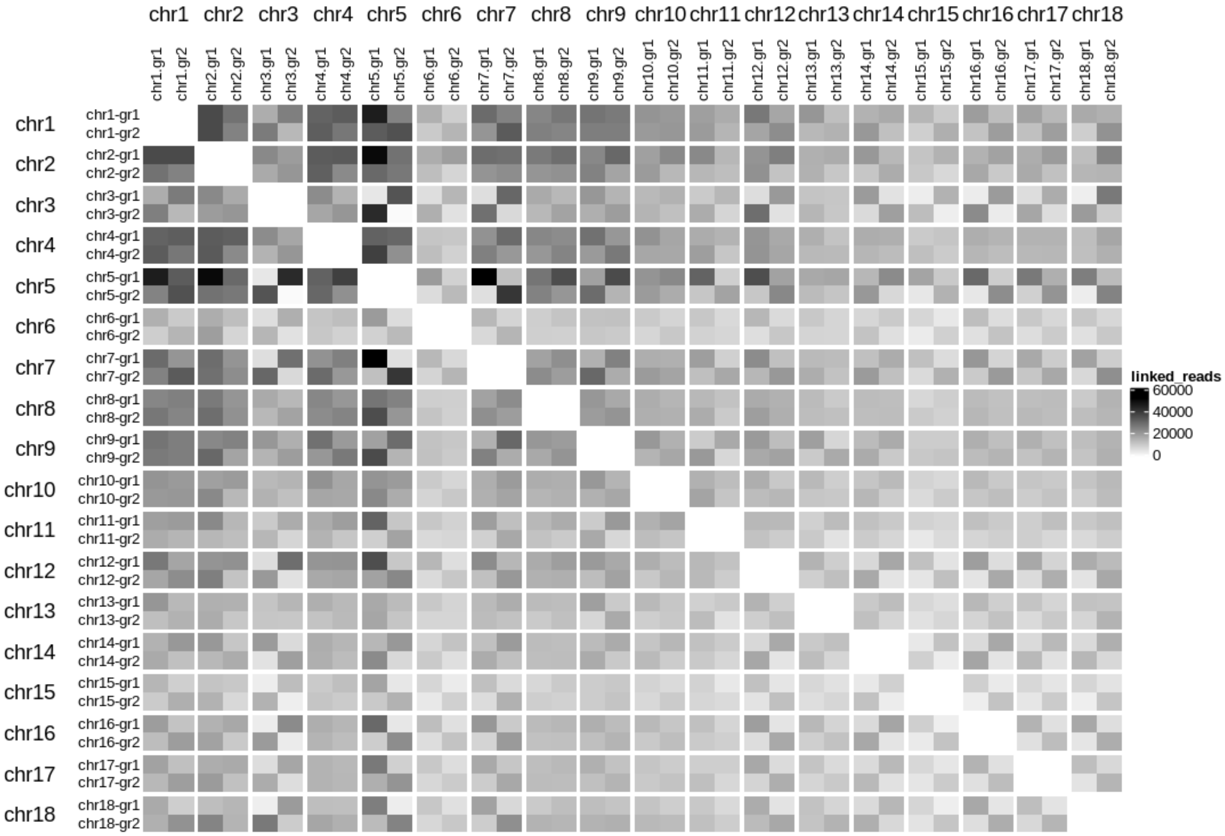

Supplementary Figure 1. Haplotype assignment was performed using Hi-C chromosome cross-linking data that indicates physical proximity in the haploid nucleus. The heatmap shows the number of Hi-C read pairs linking different chromosomes that were used for assigning chromosome assemblies to distinct haplotypes.

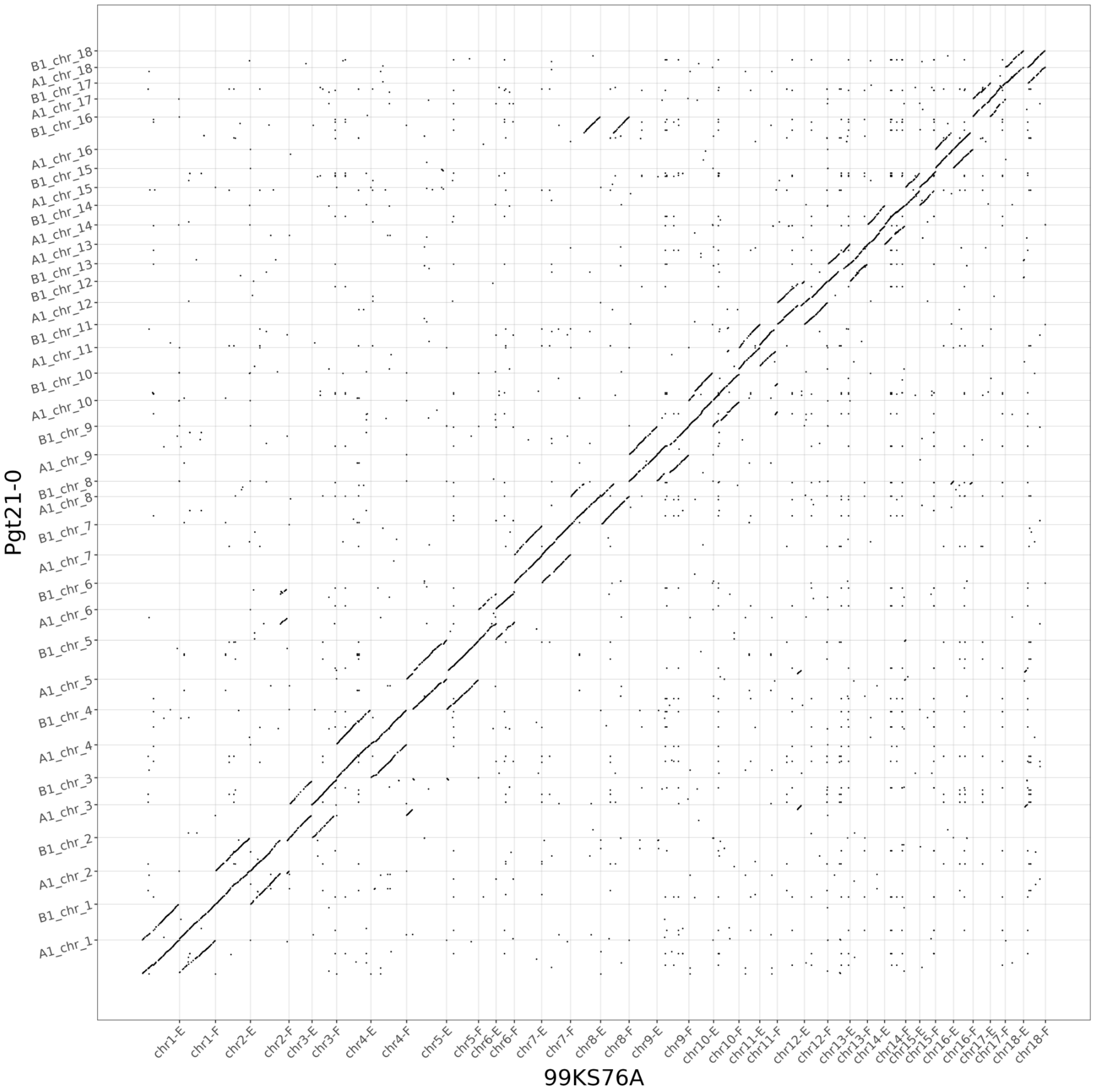

Supplementary Figure 2. Alignment of the 99KS76A-1 chromosome-scale scaffolds against the Pgt21-0 chromosomes.

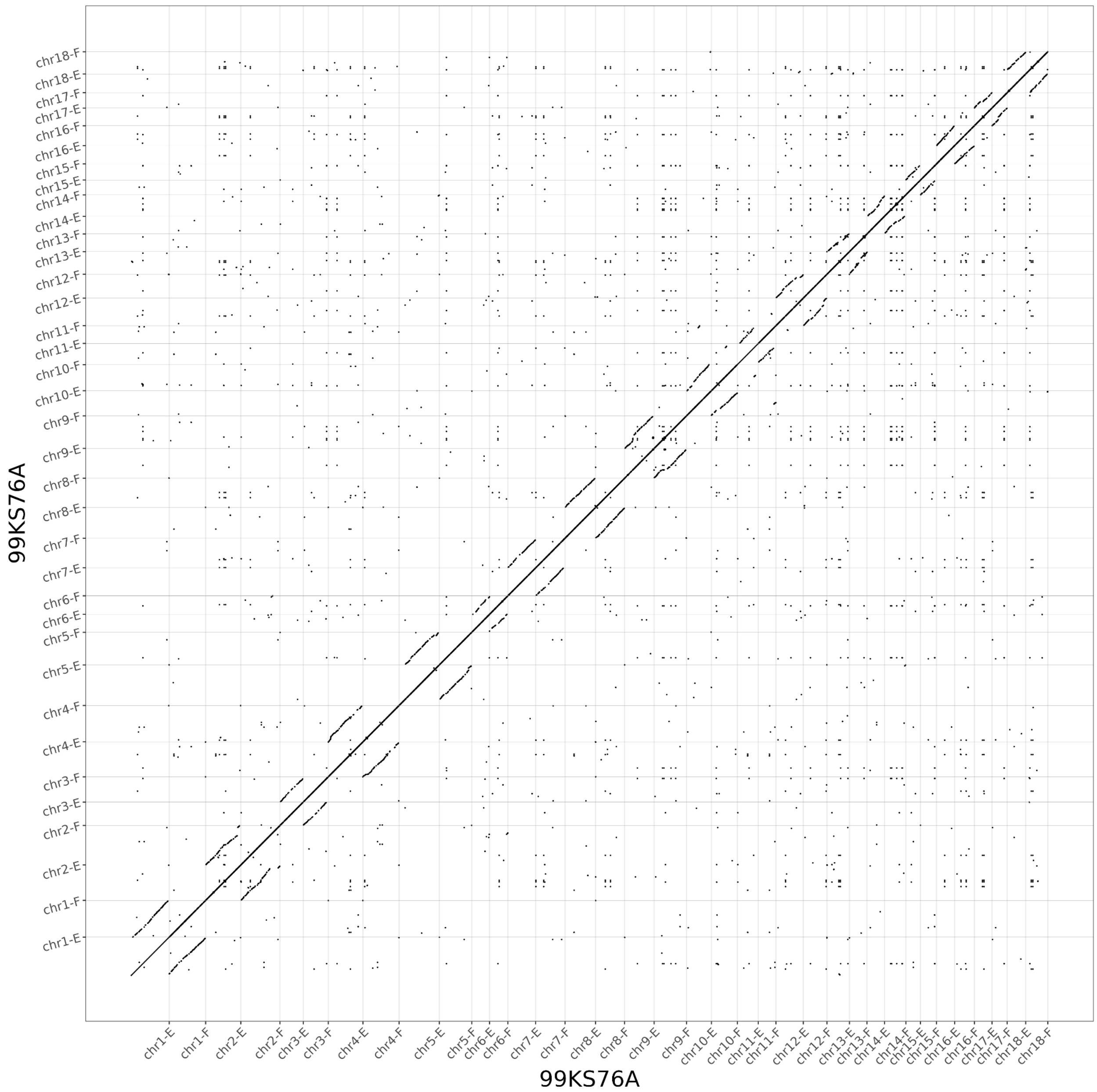

Supplementary Figure 3. The MUMmer alignment of the 99KS76A-1 scaffolds against itself.

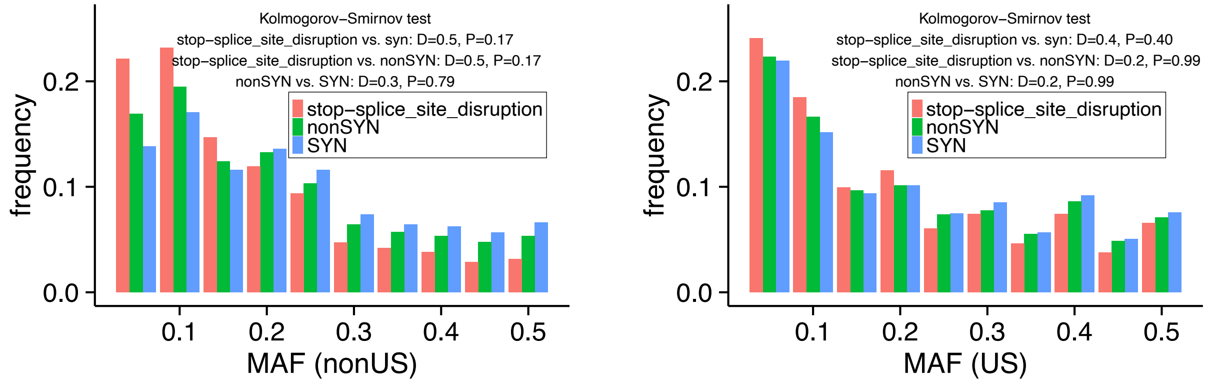

Supplementary Figure 4. MAF (minor allele frequency) of putative strong effect, non-synonymous and synonymous SNPs in the populations from the US and outside of the US. Two-sided Kolmogorov-Smirnov test was applied to compare MAF spectra between these two types of SNPs.

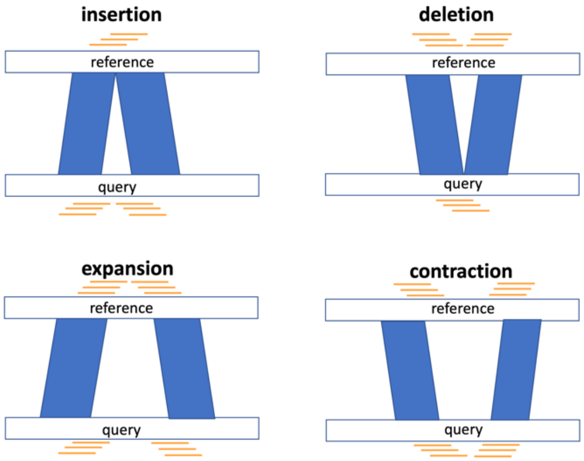

Supplementary Figure 5. Detection of structural variants (SV) types using diagnostic k-mers. The diagnostic k-mers spanning the SV boundaries are shown in orange. The homologous regions in the reference and query genomes showing sequence similarity (blue) are shown for four structural variant types: insertion, deletion, expansion and contraction.

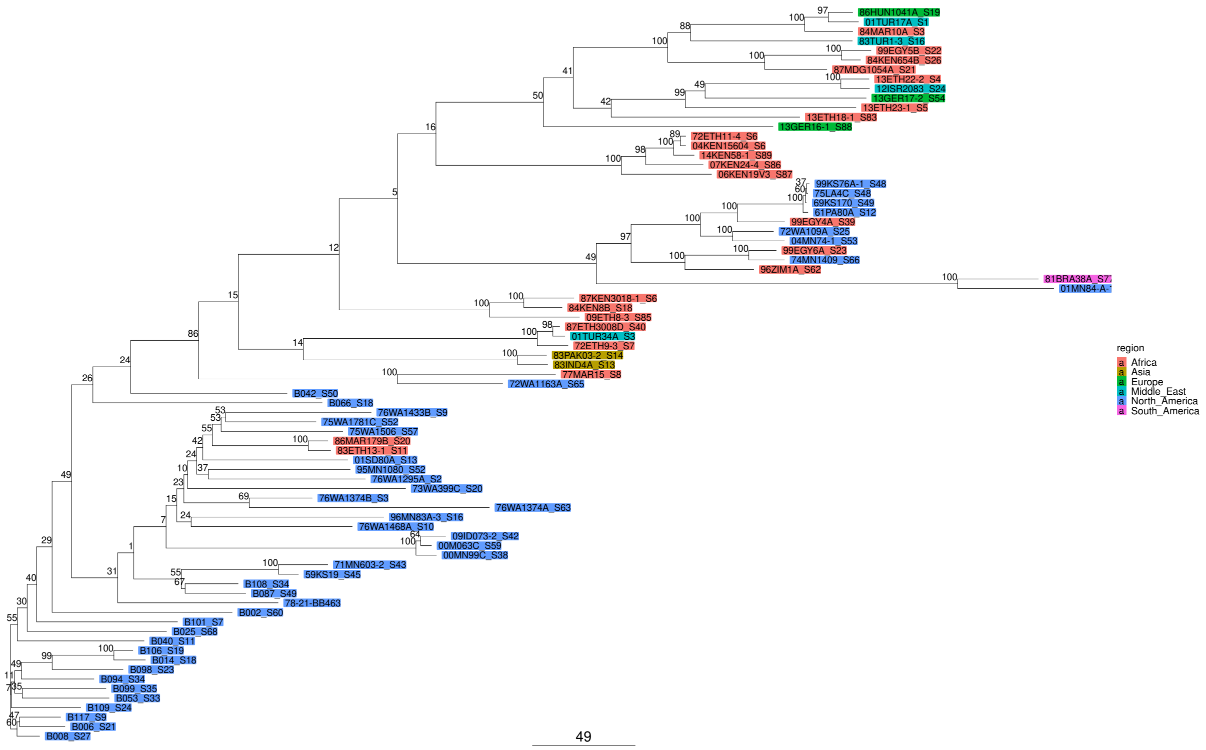

Supplementary Figure 6. Neighbor-joining phylogenetic tree based on the genotypes of structural variants generated for the Pgt panel.

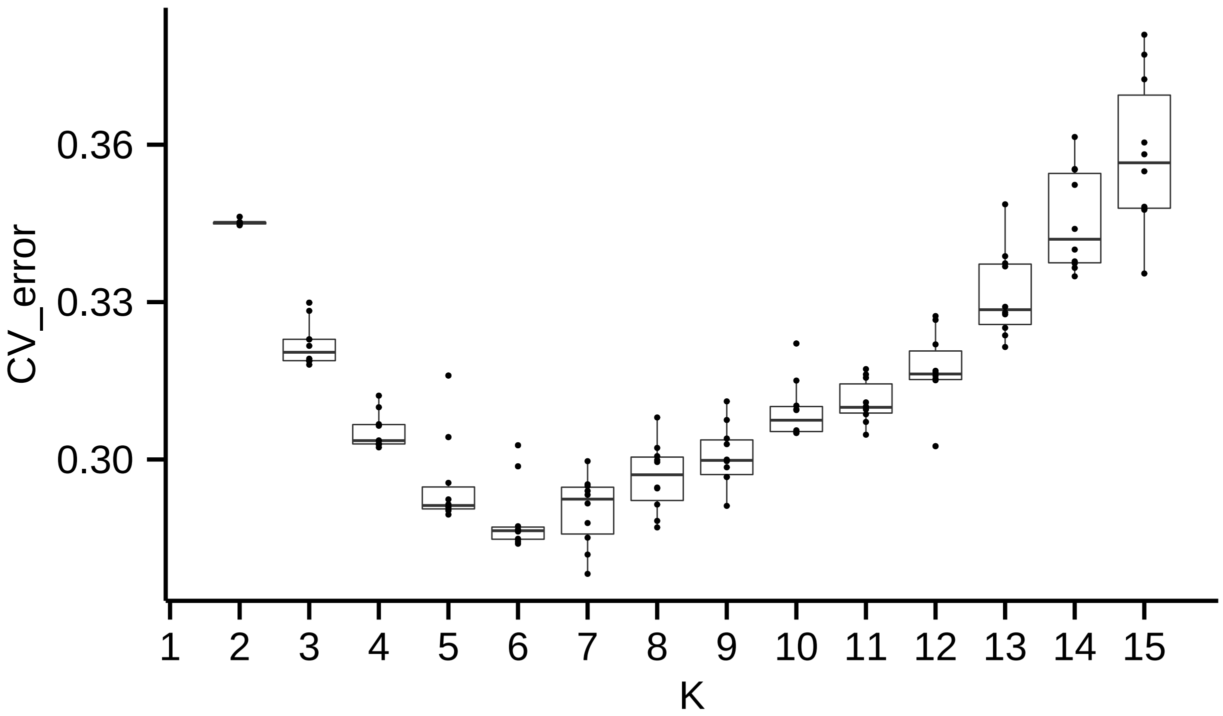

Supplementary Figure 7. Five-fold cross-validation error from the Admixture model for K ranging from 2 to 15 (10 replications for each K value). Box shows the median and interquartile ranges (IQR). The end of the top line is the maximum or the third quartile (Q) + 1.5× IQR. The end of the bottom line denotes either the minimum or the first Q − 1.5× IQR. The dots are either more than third Q + 1.5× IQR or less than first Q − 1.5× IQR.

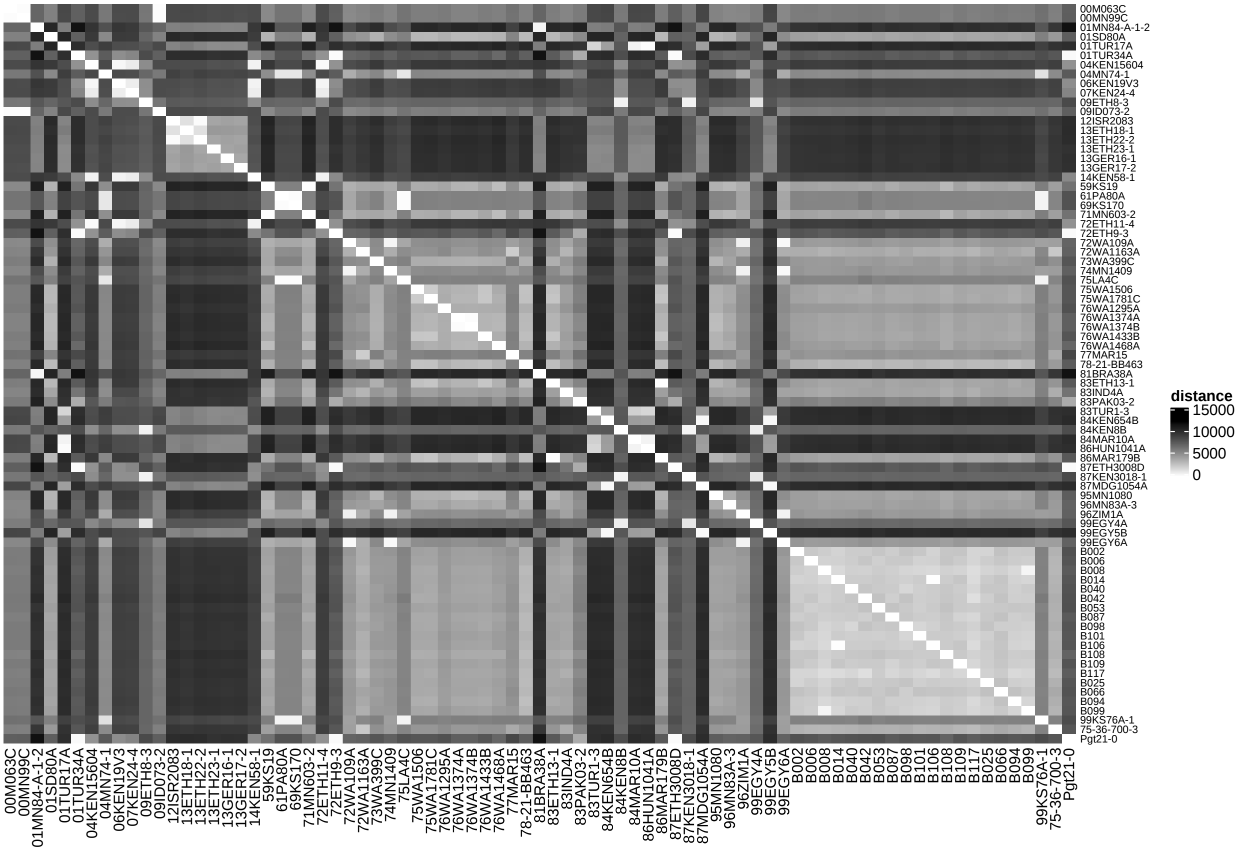

Supplementary Figure 8. Pairwise comparison of genetic distance between each isolate. Darker shades represent greater distance.

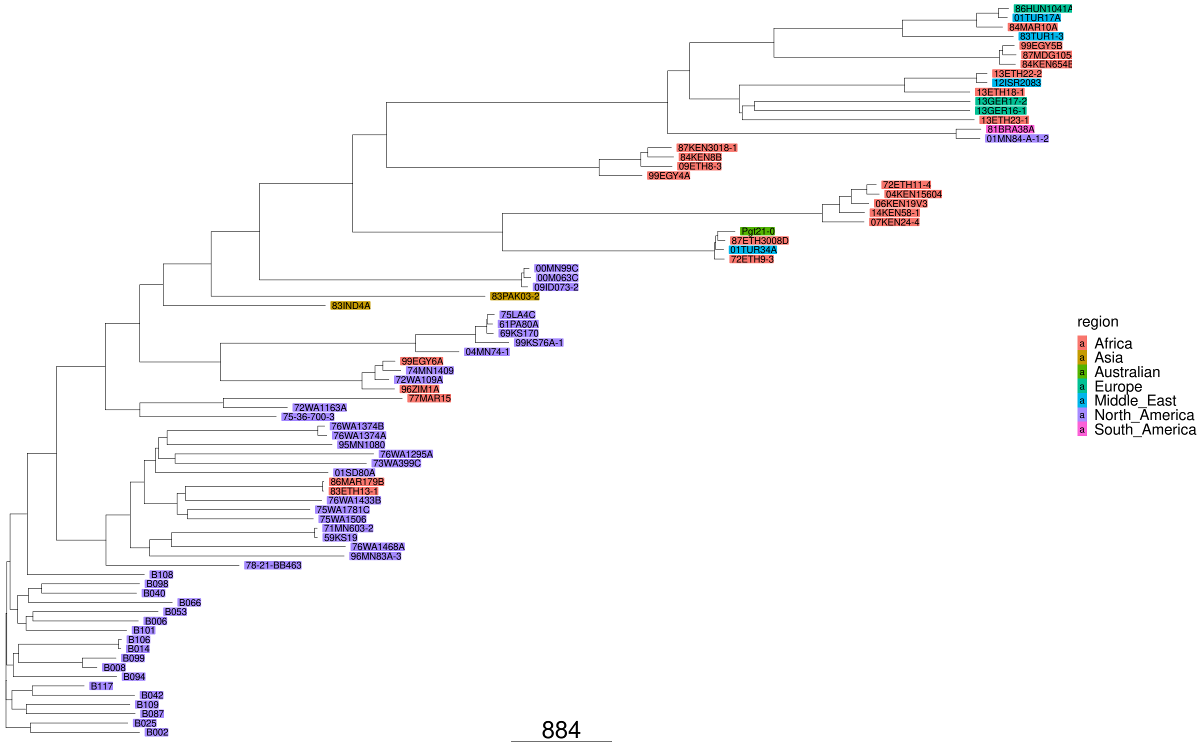

Supplementary Figure 9. Neighbor-joining phylogenetic tree based on 20K randomly selected SNPs generated for the Pgt panel with scale bar.

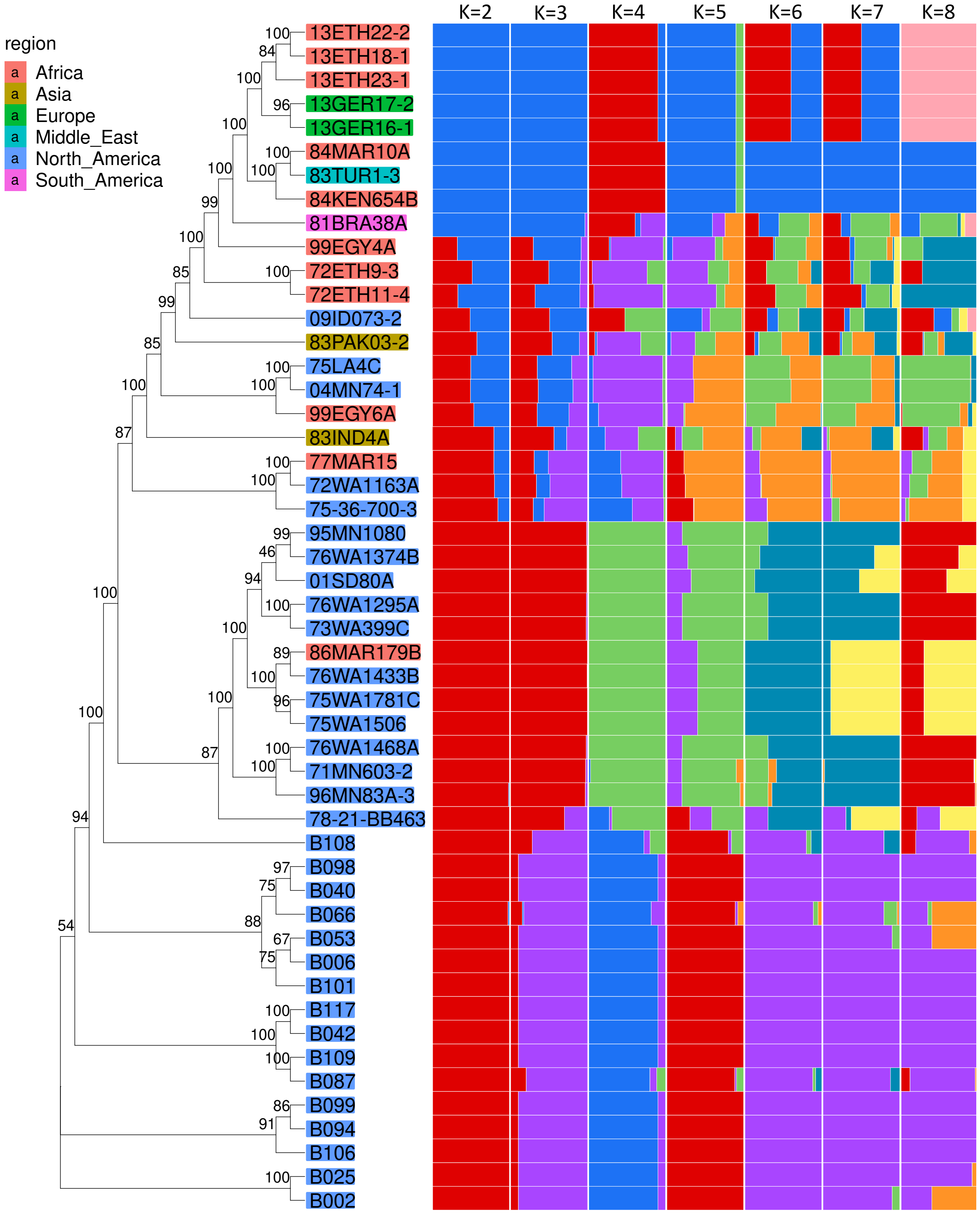

Supplementary Figure 10. Neighbor-joining phylogenetic tree and population structure for a subset of Pgt panel which includes only one isolate for each asexual lineage. Asexual lineage was defined if the divergence is smaller than Ug99 lineage.

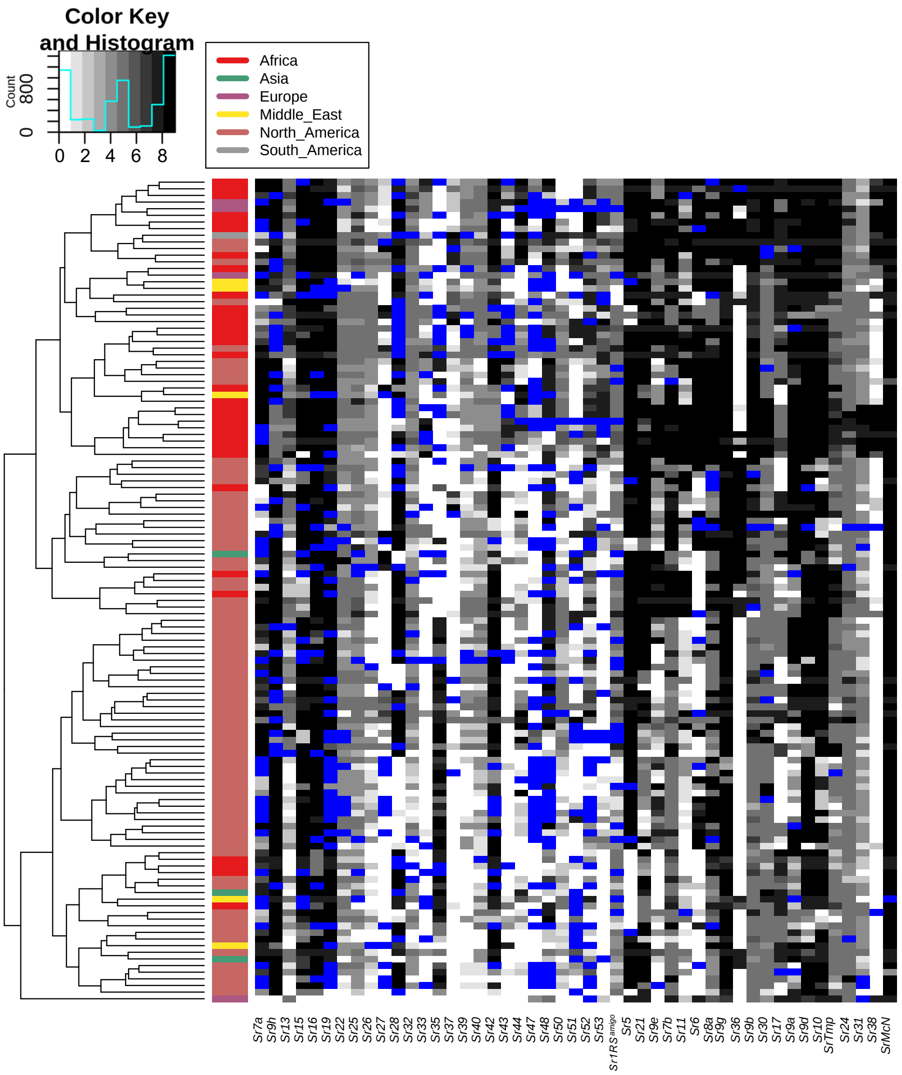

Supplementary Figure 11. Pathotype profile on 24 resistance genes of the Pgt diversity panel with the greyscale ranging from 0 (white - least compatible) to 9 (black - most compatible), missing values are colored with blue. Top left sidebar shows colors representing different geographical regions (Africa, North America, Europe, Middle East, Asia, and South America).

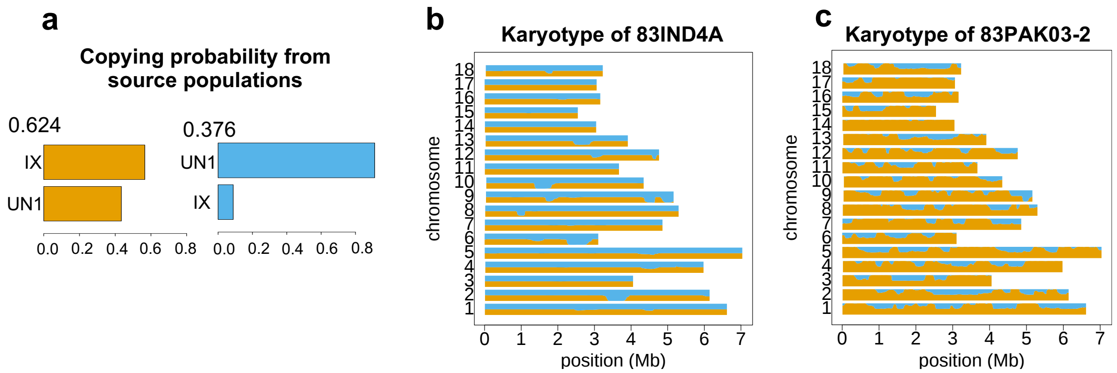

Supplementary Figure 12. Estimates of expected local ancestry for clade UN2, modeled as a two-way admixture event ($r^2 = 0.82$). **a.** The probability of copying from two donor clades IX and UN1. Colors correspond to ancestries in each donor clade. **b-c.** Genome-wide estimates of clade UN2 isolates in two donor source populations.

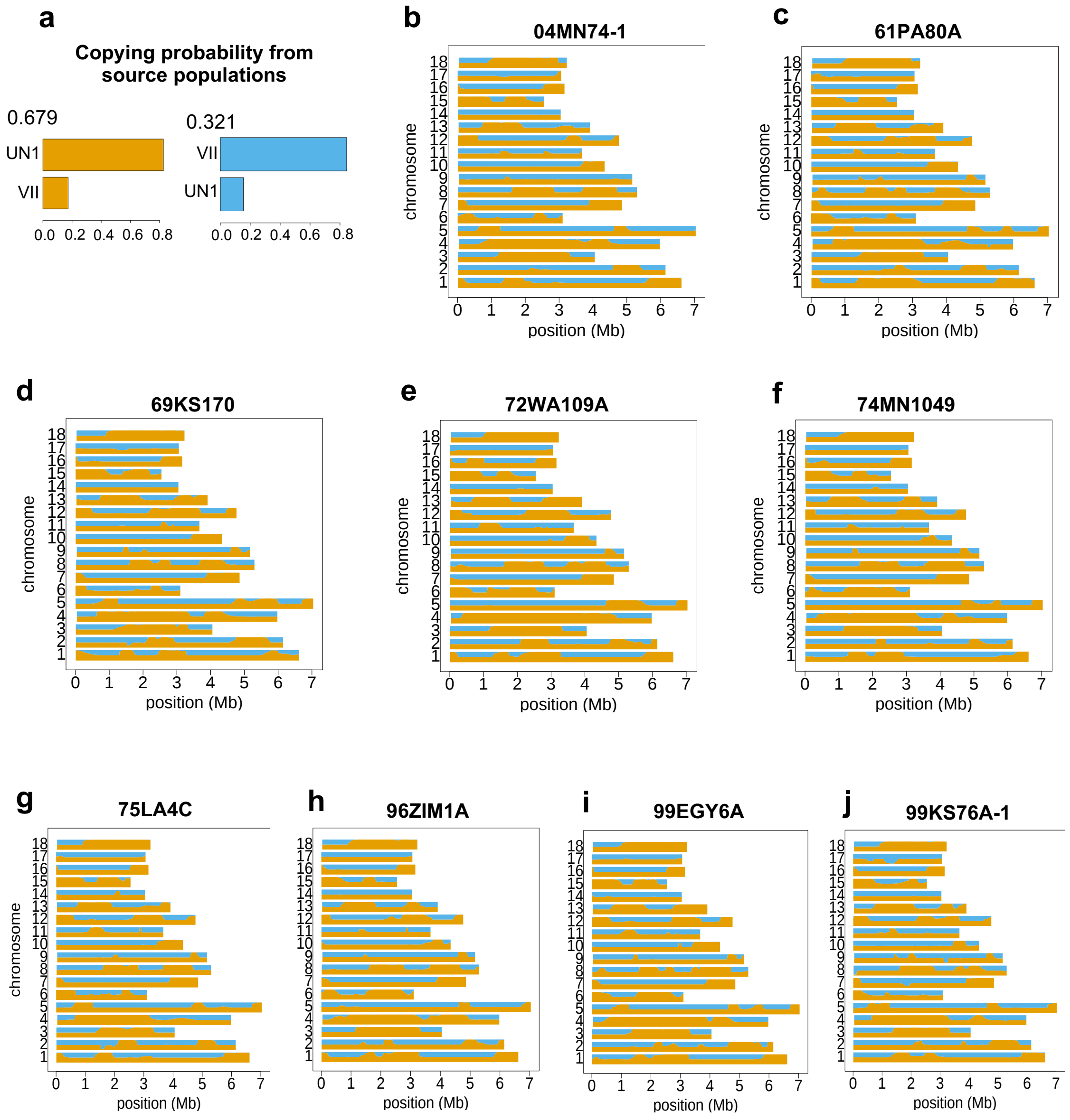

Supplementary Figure 13. Estimates of expected local ancestry for clade VI-A, modeled as a two-way admixture event ($r^2 = 0.87$). **a.** The probability of copying from two donor clades VII and UN1. Colors correspond to ancestries in each donor clade. **b-j.** Genome-wide estimates of clade VI-A isolates in two donor source populations.

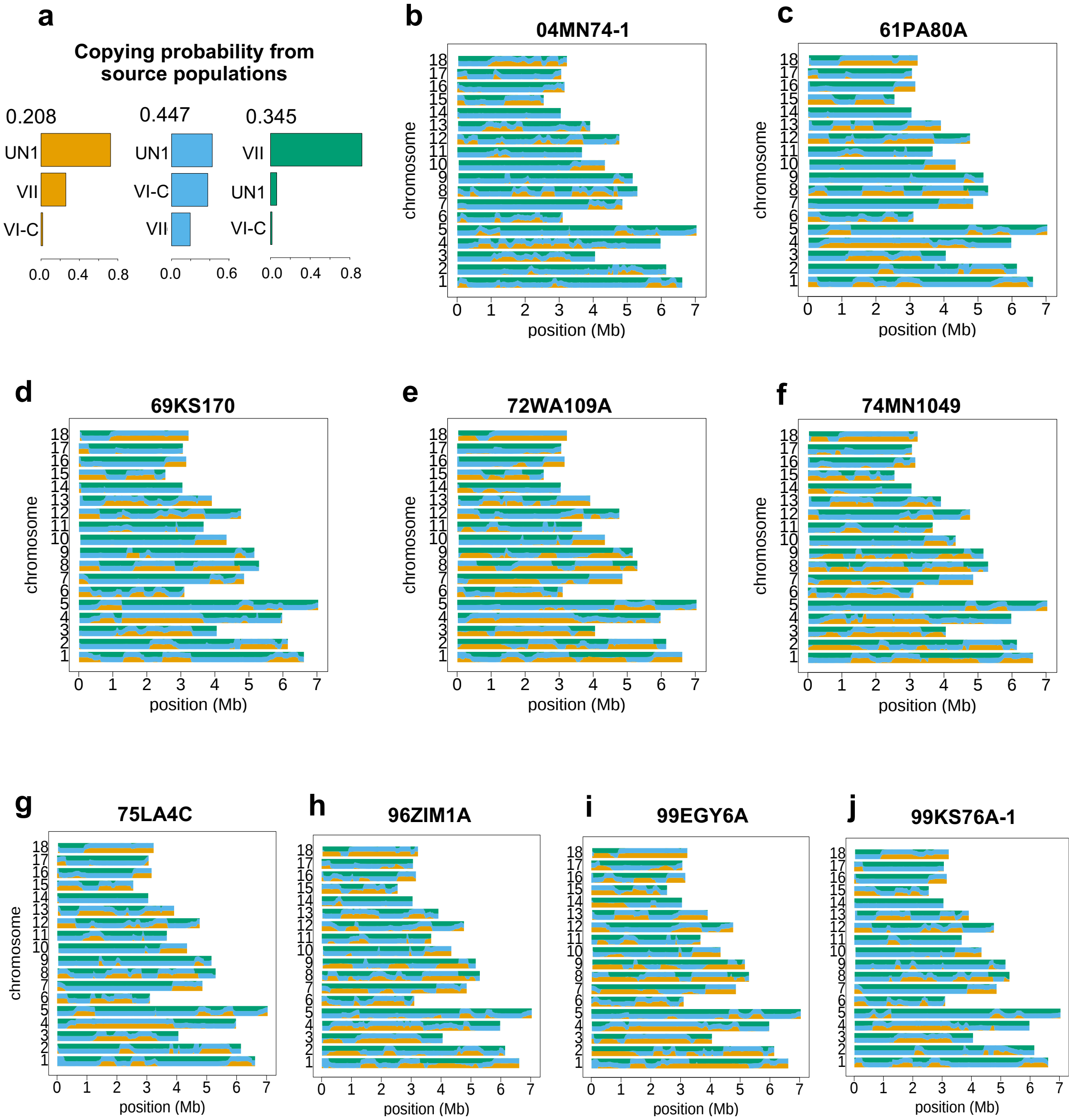

Supplementary Figure 14. Estimates of expected local ancestry for clade VI-A, modeled as a three-way admixture event ($r^2 = 0.78$). **a.** The probability of copying from three donor clades VII, UN1 and VI-C. Colors correspond to ancestries in each donor clade. **b-j.** Genome-wide estimates of clade VI-A isolates in three donor source populations.

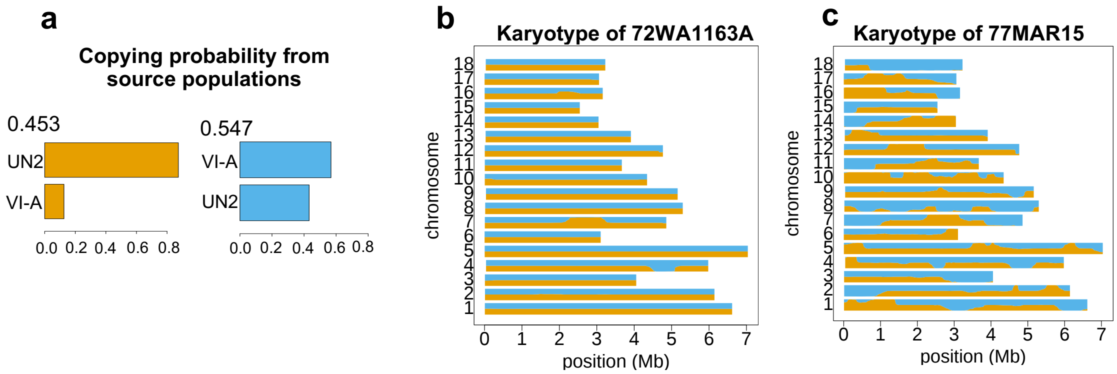

Supplementary Figure 15. Estimates of expected local ancestry for clade UN1, modeled as a two-way admixture event ($r^2 = 0.85$). **a.** The probability of copying from two donor clades VI-A and UN2. Colors correspond to ancestries in each donor clade. **b-c.** Genome-wide estimates of clade UN1 isolates in two donor source populations.

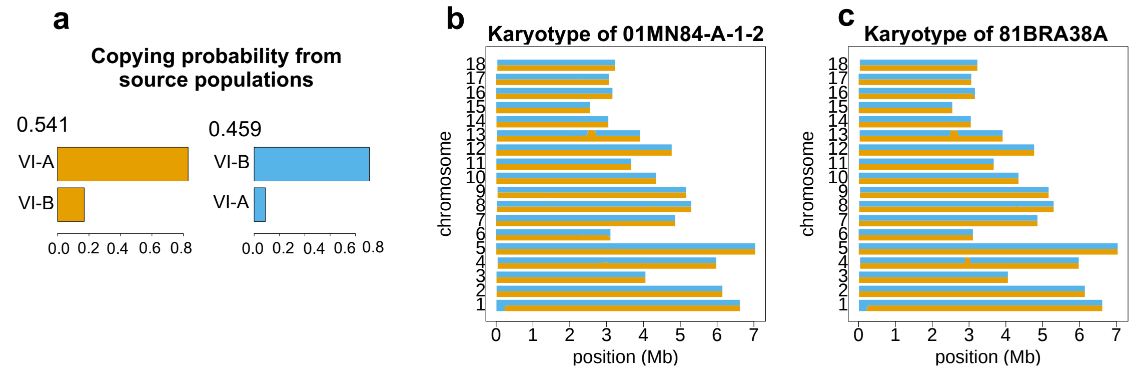

Supplementary Figure 16. Estimates of expected local ancestry for clade VII, modeled as a two-way admixture event ($r^2 = 0.88$). **a.** The probability of copying from two donor clades VI-A and VI-B. Colors correspond to ancestries in each donor clade. **b-c.** Genome-wide estimates of clade VII isolates in two donor source populations.

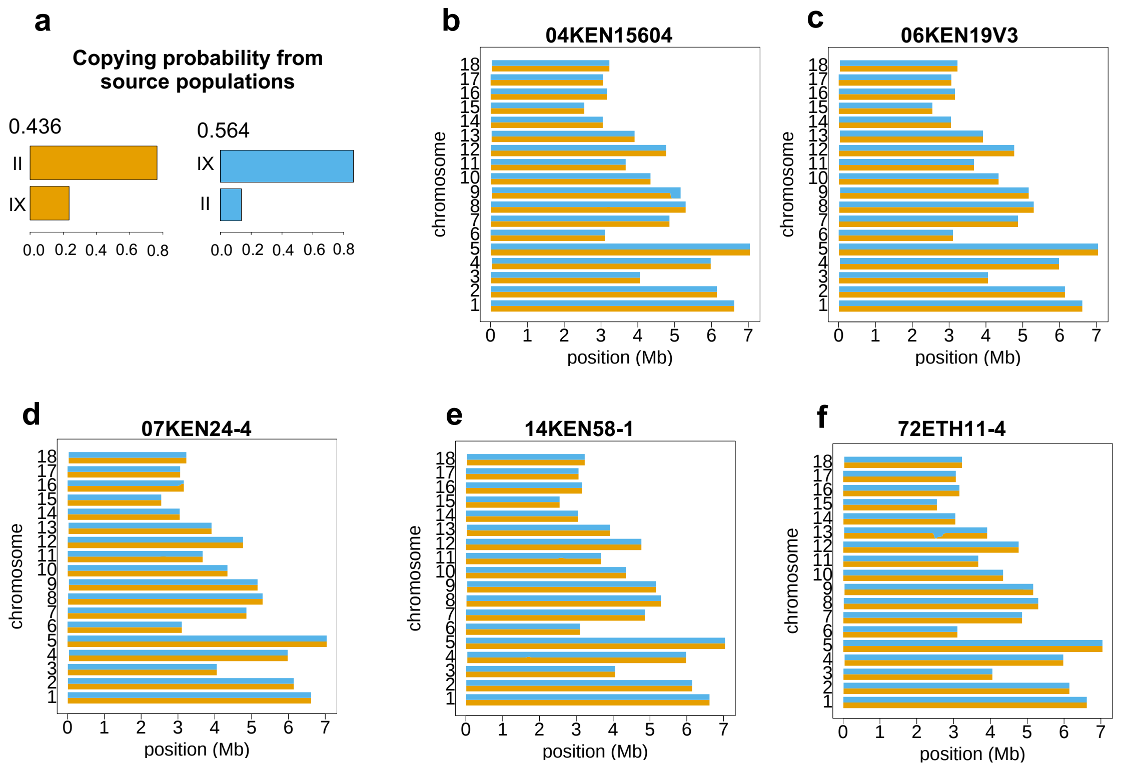

Supplementary Figure 17. Estimates of expected local ancestry for clade I (Ug99 group), modeled as a two-way admixture event ($r^2 = 0.72$). **a.** The probability of copying from two donor clades II and IX. Colors correspond to ancestries in each donor clade. **b-f.** Genome-wide estimates of clade I isolates in two donor source populations.

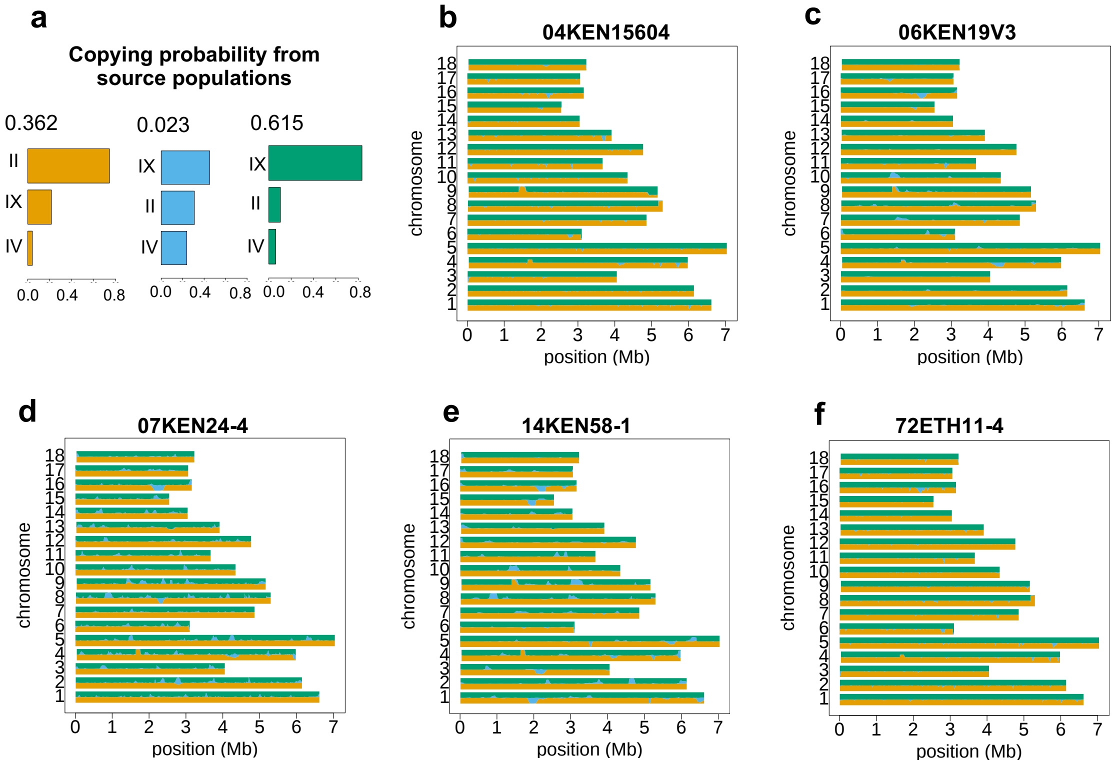

Supplementary Figure 18. Estimates of expected local ancestry for clade I (Ug99 group), modeled as a three-way admixture event ($r^2 = 0.35$). **a.** The probability of copying from three donor clades II, IX and IV. Colors correspond to ancestries in each donor clade. **b-f.** Genome-wide estimates of clade I isolates in three donor source populations.

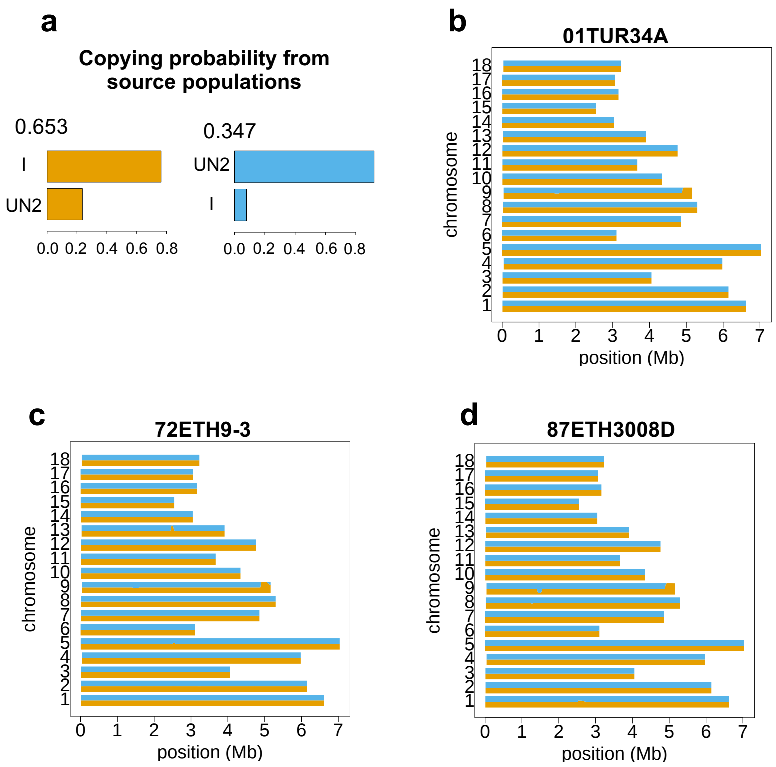

Supplementary Figure 19. Estimates of expected local ancestry for clade IX, modeled as a two-way admixture event ($r^2 = 0.78$). **a.** The probability of copying from two donor clades I and UN2. Colors correspond to ancestries in each donor clade. **b-d.** Genome-wide estimates of clade IX isolates in two donor source populations.

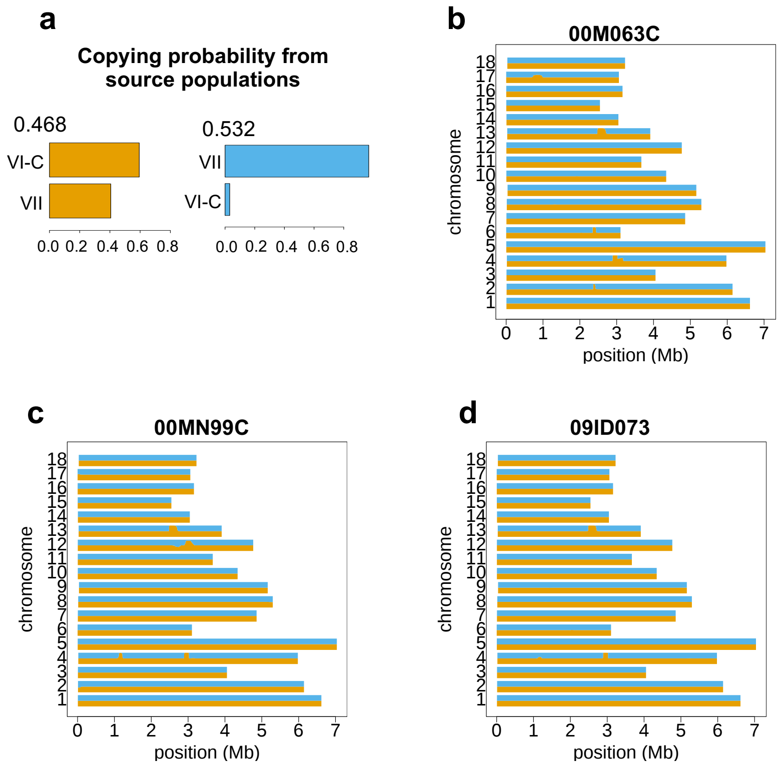

Supplementary Figure 20. Estimates of expected local ancestry for clade VI-B, modeled as a two-way admixture event ($r^2 = 0.86$). **a.** The probability of copying from two donor clades VI-C and VII. Colors correspond to ancestries in each donor clade. **b-d.** Genome-wide estimates of clade VI-B isolates in two donor source populations.

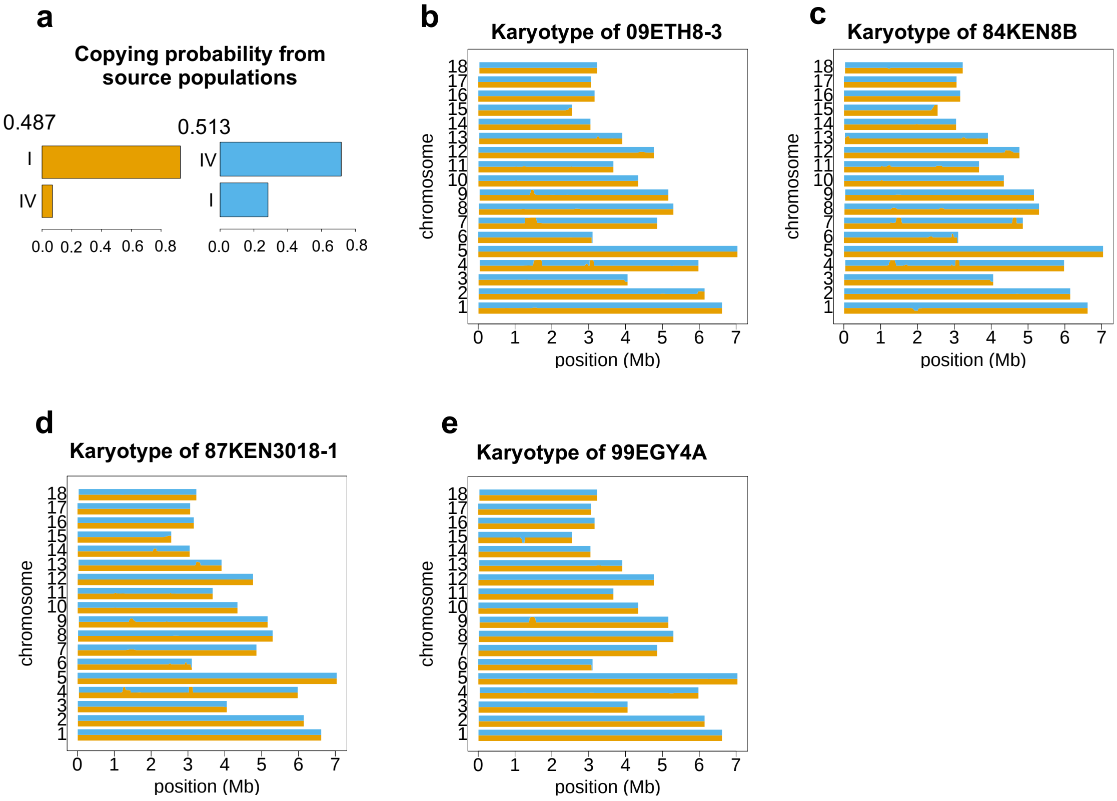

Supplementary Figure 21. Estimates of expected local ancestry for clade II, modeled as a two-way admixture event ($r^2 = 0.69$). **a.** The probability of copying from two donor clades I and IV. Colors correspond to ancestries in each donor clade. **b-e.** Genome-wide estimates of clade II isolates in two donor source populations.

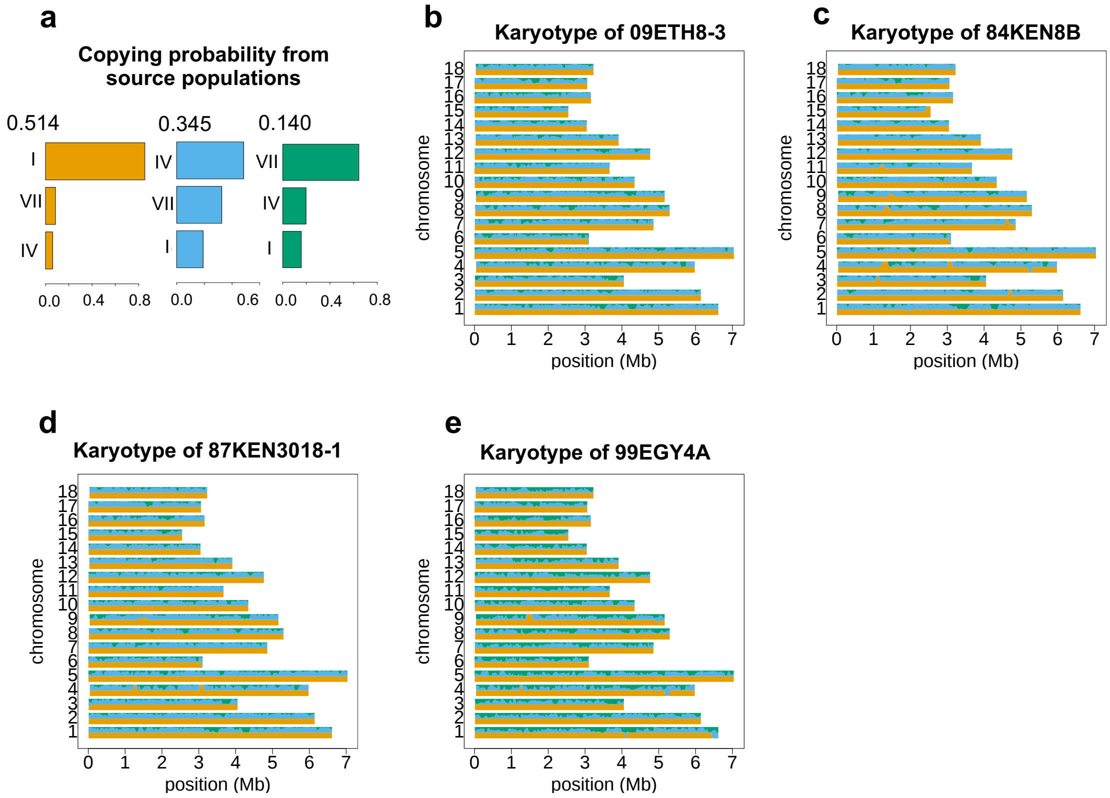

Supplementary Figure 22. Estimates of expected local ancestry for clade II, modeled as a three-way admixture event ($r^2 = 0.43$). **a.** The probability of copying from three donor clades I, IV and VII. Colors correspond to ancestries in each donor clade. **b-e.** Genome-wide estimates of clade II isolates in three donor source populations.

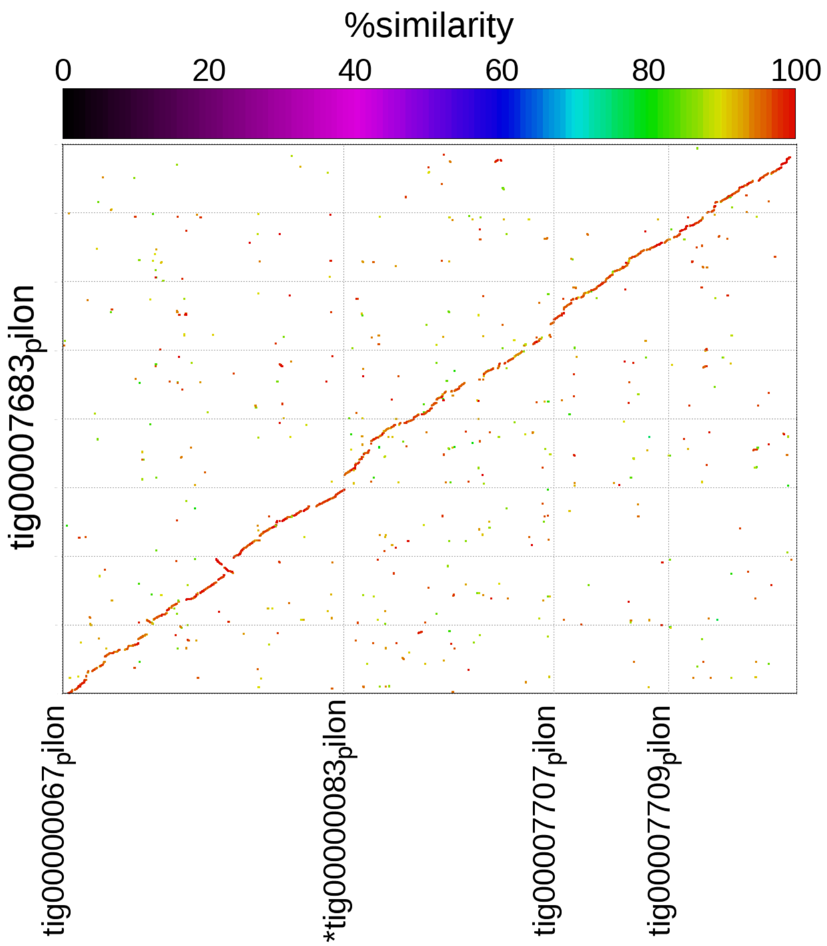

Supplementary Figure 23. Example of bin assignment to resolve the two haplotypes in the 99KS76A-1 assembly. The tig00007683_pilon contig shows a high level of similarity to contigs tig00007709_pilon, tig00007707_pilon, tig00000083_pilon and tig00000067_pilon in Bin28. See Supplementary Table 1.

Supplementary Tables

Supplementary Table 1. Example of bin assignments to resolve two haplotypes. Homologous matching contigs (tig00007683_pilon vs tig00007709_pilon, tig00007707_pilon, tig00000083_pilon and tig00000067_pilon) were grouped into the same bin.

Reference assembly contig	Matching contigs	# of shared genes
tig00007683_pilon	tig00007709_pilon	40
	tig00007707_pilon	50
	tig00000083_pilon	61
	tig00000067_pilon	99

Supplementary Table 2. The 99KS76A-1 genome assembly metrics obtained at different assembly steps.

	**Canu**	**Pilon**	**ALLHiC**
Number of contigs	1162	1162	1021
Largest contig, bp	5,440,655	5,536,814	7,041,308
Smallest contig, bp	1,020	1,020	1,020
Total length, bp	178,353,835	180,987,563	181,001,863
N50, bp	1,329,390	1,345,248	4,517,833

Supplementary Table 3. Comparison of the completeness of the 99KS76A-1, Pgt21-0 and Ug99 assembly (based on BUSCO).

**Classes of genes**	**99KS76A-1**	**99KS76A-1 (E)**	**99KS76A-1 (F)**	**Pgt21-0**	**Ug99**
Complete and single-copy	346	1520	1503	168	188
Complete and duplicated	1266	33	26	1436	1398
Fragmented	22	20	22	20	32
Missing	130	191	213	140	146

Supplementary Table 4. Summary of predicted genes, e.g. length of the genes, distance between genes, etc.

	Total number of genes	Mean	Max	Min	STD	SEM
Gene cds length	22,974	1174.6	11316.0	267.0	929.7	6.1
Gene genomic region length		2154.0	499325.0	297.0	8000.3	52.8
Gene interval		5645.1	358876.0	1.0	10448.0	70.8

Supplementary Table 5. Summary of predicted effector-encoding genes.

	Total number of effectors	Mean	Max	Min	STD	SEM
Effector cds length	3952	956.7	6285.0	297.0	686.2	10.9
Effector genomic region length		1933.3	499325.0	300.0	11076.8	176.2

Supplementary Table 6. The functional effects of SNPs on a gene.

Tentative functional effects of SNPs	Number of SNPs	Proportion
**HIGH**	9,065	0.17%
**LOW**	306,610	5.59%
**MODERATE**	191,107	3.49%
**MODIFIER**	4.976,207	90.76%

Supplementary Table 7. Number of SNPs with different functional effects on coding sequence.

SNP types	Number of SNPs	Proportion
**MISSENSE**	187,704	40.53%
**NONSENSE**	2,712	0.59%
**SILENT**	272,742	58.89%

Supplementary Table 8. Distribution of SNPs across annotated features in the Pgt genome.

SNP locations	Number of SNPs	Proportion
**DOWNSTREAM**	1,591,321	29.023%
**EXON**	468,186	8.539%
**INTERGENIC**	1,430,242	26.085%
**INTRON**	271,023	4.943%
**SPLICE_SITE_ACCEPTOR**	485	0.009%
**SPLICE_SITE_DONOR**	619	0.011%
**SPLICE_SITE_REGION**	32,090	0.585%
**UPSTREAM**	1,583,233	28.875%
**UTR_3_PRIME**	59,913	1.093%
**UTR_5_PRIME**	45,843	0.836%

Supplementary Table 9. Number of synonymous and non-synonymous SNPs in the Pgt genome.

SNP types	Number of SNPs	Proportion
**SYNONYMOUS_CODING**	272,321	4.935%
**NON_ SYNONYMOUS_CODING**	187,085	3.391%

Supplementary Table 10. Summary of nucleotide diversity statistics per gene.

	**Mean**	**SEM**
Gene regions (remove TE)	0.0034	2.97e-05
Exons (remove TE)	0.0030	1.87e-05
Introns (remove TE)	0.0045	3.64e-05
Syn (remove TE)	0.0010	1.23e-05
Non-syn (remove TE)	0.0015	1.65e-05

Supplementary Table 11. Distribution of synonymous, nonsynonymous and stop codon and splice site disruption causing mutations between the effectors and non-effectors.

Types of SNP variants	Effectors	Non-effectors
Nonsynonymous	33420	153653
Synonymous	35779	236522
Stop and splice site disruption	535	3172
Stop and splice site disruption/syn	0.0150	0.0134
Ratio of nonsyn/syn SNPs	0.9341	0.6496
Stop and splice site disruption/nonsyn	0.0160	0.0206

Supplementary Table 12. Groups of isolates from distinct geographic regions used for analyses of selective sweeps, Tajima's D, LD and genetic differentiation tests with the purpose of identifying regions showing signatures of selection and local adaptation.

**Isolated groups**	
**US isolates**	**78-21-BB463 96MN83A-3 76WA1468A 59KS19 71MN603-2 95MN1080 73WA399C 76WA1295A 76WA1374A 76WA1374B 01SD80A 76WA1433B 75WA1506 75WA1781C 72WA1163A**
**Non-US isolates**	**09ETH8-3 84KEN8B 87KEN3018-1 07KEN24-4 14KEN58-1 06KEN19V3 04KEN15604 72ETH11-4 81BRA38A 13GER17-2 13ETH23-1 13GER16-1 13ETH18-1 12ISR2083 13ETH22-2 84KEN654B 87MDG1054A 99EGY5B 83TUR1-3 84MAR10A 01TUR17A 86HUN1041A**

Supplementary Table 13. List of effector-encoding genes overlapping with the selective sweep regions identified by combining the results of XP-CLR and SweeD scans.

asmbl_179.p1	asmbl_4405.p1	asmbl_24437.p1
asmbl_259.p1	asmbl_4579.p1	asmbl_24228.p1
asmbl_277.p1	asmbl_4785.p1	asmbl_24217.p1
asmbl_440.p1	asmbl_4911.p1	asmbl_24078.p1
asmbl_503.p1	asmbl_6138.p1	asmbl_23950.p1
asmbl_815.p1	asmbl_6140.p1	asmbl_23718.p1
asmbl_817.p1	asmbl_6313.p1	asmbl_23646.p1
asmbl_885.p1	asmbl_7998.p1	asmbl_27455.p1
asmbl_1104.p1	asmbl_8048.p1	asmbl_27290.p3
asmbl_1356.p1	asmbl_8409.p1	asmbl_27265.p1
asmbl_1448.p1	asmbl_8498.p2	asmbl_29626.p1
asmbl_1455.p1	asmbl_8548.p1	asmbl_29751.p1
asmbl_1485.p1	asmbl_9883.p1	asmbl_29857.p1
asmbl_28677.p1	asmbl_9939.p1	asmbl_29889.p1
asmbl_28244.p1	asmbl_15728.p1	asmbl_29890.p1
asmbl_28169.p1	asmbl_15896.p1	asmbl_30417.p1
asmbl_28038.p1	asmbl_15961.p1	asmbl_30503.p1
asmbl_27892.p1	asmbl_16122.p1	asmbl_30504.p1
asmbl_27889.p1	asmbl_16336.p1	asmbl_30730.p1
asmbl_27833.p1	asmbl_16681.p1	asmbl_30837.p1
asmbl_39810.p1	asmbl_18924.p1	asmbl_30915.p1
asmbl_39976.p1	asmbl_18927.p1	asmbl_35403.p1
asmbl_40077.p1	asmbl_19091.p1	asmbl_35297.p1
asmbl_40199.p1	asmbl_19254.p1	asmbl_35294.p1
asmbl_40493.p1	asmbl_23102.p1	asmbl_34824.p1
asmbl_41578.p1	asmbl_23096.p1	asmbl_34699.p1
asmbl_41790.p1	asmbl_23022.p1	asmbl_34453.p1
asmbl_41800.p1	asmbl_23020.p1	asmbl_34451.p1
asmbl_42245.p1	asmbl_22960.p1	asmbl_34353.p1
asmbl_44178.p1	asmbl_22920.p3	asmbl_34295.p1
asmbl_44256.p1	asmbl_22919.p1	asmbl_34174.p1
asmbl_44262.p1	asmbl_22895.p1	asmbl_33972.p1
asmbl_44372.p1	asmbl_22812.p1	asmbl_37381.p1
asmbl_44496.p1	asmbl_22741.p1	asmbl_37412.p1
asmbl_44659.p1	asmbl_22132.p1	asmbl_37417.p1
asmbl_44666.p1	asmbl_21807.p1	asmbl_37421.p1

asmbl_44692.p1	asmbl_25010.p1	asmbl_37423.p1
asmbl_44749.p1	asmbl_24886.p1	asmbl_37427.p1
asmbl_44752.p1	asmbl_24884.p1	asmbl_37960.p1
asmbl_45026.p1	asmbl_24675.p1	asmbl_38058.p1

Supplementary Table 14. Genome wide R^2 of 2-way and 3-way MOSAIC analysis for the admixed Pgt clades using remaining all clades as references. Most clades show higher R^2 for 2-way admixture model compared to 3-way admixture model, except three clades. For clade VI-A, R^2 of the 3-way model was higher than that of the 2-way model. For clades I and II, R^2 values for the 2- and 3-way models were similar. For these three clades, MOSAIC analyses were repeated using only subsets of donor populations.

	**I**	**VI-A**	**VII**	**IX**	**VI-B**	**UN1**	**UN2**	**II**
2-way	0.37	0.71	0.60	0.72	0.87	0.89	0.92	0.75
3-way	0.37	0.76	0.58	0.41	0.49	0.79	0.84	0.75

Supplementary Table 15.

Admixed clade	Source 1	Source 2	Num. *Sr* genes effective against Pgt from source 1 and source 2	Num. *Sr* genes not effective against Pgt from admixed clade
VII	VIB	VIA	15	3
II	IV	I	8	1
I (Ug99)	IX	II	13	4
IX	I	UN2	9	0
VIB	VII	VIC	10	0

Supplementary Table 16. The disease resistance scores* of F_2 isolates on six *Sr* genes compared to two parental isolates.

Pgt isolate	Comment	*Sr7b*	*Sr11*	*Sr6*	*Sr9b*	*Sr30*	*Sr24*
7a.rep1	Parent_7a	5.33	1.33	0	5	5	5
7a.rep2	Parent_7a	5	5	0	5	5	5
4a.rep1	Parent_4a	5	1	1	5	4	4
4a.rep2	Parent_4a		5	1			5
B002.rep1	F2	9	0.67	8.33	6	5	5
B002.rep2	F2	5.33	1.33	9	5		5
B006.rep1	F2	5	1.33	0	5	9	4.67
B008.rep1	F2	5	0	9	5	1.33	5
B013.rep1	F2	5	0.67	0	6.67		6.67
B013.rep2	F2	5	1.33	9	5	5	5
B014.rep1	F2	5	9		8	8	6
B019.rep1	F2	5.33	0	9	6	5	5.33
B019.rep2	F2	8.33	2.67	9	5	5	4.67
B025.rep1	F2	5	4	3.67	5	5	5
B025.rep2	F2	5.33	0.67	6.33	3	4.67	5
B040.rep1	F2	5	1.33	0.67	5	8.33	5
B053.rep1	F2	5	1.33	8	5.33	5	5.33
B053.rep2	F2	5	0	0	5	5	5
B066.rep1	F2	5	1.33	3.67	5	9	5
B078.rep1	F2	8	0.33	8.33	5.33	5	5
B087.rep1	F2	5	0	9	6.67	5	5.33

B094.rep1	F2	5	1.33	9	9	5	5
B098.rep1	F2	9	1.33	0.33	6.67		4
B099.rep1	F2	5	1.33	0.33	7.33	9	5
B101.rep1	F2	5.33	4	0	5.33	5	5.33
B101.rep2	F2	5	4		5	5	5
B106.rep1	F2	5	5	0	8	6	8
B108.rep1	F2	5	0	0.67	5.33	5	5.33
B109.rep1	F2	5.33	1.33	8	5	5	4
B117.rep1	F2	5	9	4	6	9	5

* 0 (least compatible) to 9 (most compatible)
