# Supplementary material for: Population genomics of Puccinia graminis f.sp. tritici highlights the role of admixture in the origin of virulent wheat rust races
Source: Nat Commun. 2022 Oct 21;13:6287. doi: 10.1038/s41467-022-34050-w (PMC9587050; doi:10.1038/s41467-022-34050-w)
Supplement: Supplementary file 3 — Description of Additional Supplementary Files [file 41467_2022_34050_MOESM3_ESM.pdf]

Description of Additional Supplementary Files

Supplementary data 1:
Predicted effector-encoding genes.

Supplementary data 2:
Pgt isolates included into the diversity analysis.

Supplementary data 3:
Effectors affected by all types (insertion, deletion, contraction and expansion) of detected SVs.

Supplementary data 4:
Genotypes of SVs (insertion and deletion) affecting effectors.

Supplementary data 5:
Pgt isolates included in MOSAIC analysis.

Supplementary data 6:
List of Pgt isolates with available virulence scores and their assignment to phylogenetic clades.
