# Supplementary material for: Population genomics of Puccinia graminis f.sp. tritici highlights the role of admixture in the origin of virulent wheat rust races
Source: Nat Commun. 2022 Oct 21;13:6287. doi: 10.1038/s41467-022-34050-w (PMC9587050; doi:10.1038/s41467-022-34050-w)
Supplement: Supplementary file 10 — Reporting Summary [file 41467_2022_34050_MOESM10_ESM.pdf]

nature portfolio

Corresponding author(s): Eduard Akhunov

Last updated by author(s): Sep 15, 2022

Reporting Summary

Nature Portfolio wishes to improve the reproducibility of the work that we publish. This form provides structure for consistency and transparency in reporting. For further information on Nature Portfolio policies, see our Editorial Policies and the Editorial Policy Checklist.

Statistics

For all statistical analyses, confirm that the following items are present in the figure legend, table legend, main text, or Methods section.

n/a	Confirmed	
☐	☒	The exact sample size (*n*) for each experimental group/condition, given as a discrete number and unit of measurement
☒	☐	A statement on whether measurements were taken from distinct samples or whether the same sample was measured repeatedly
☐	☒	The statistical test(s) used AND whether they are one- or two-sided<br>*Only common tests should be described solely by name; describe more complex techniques in the Methods section.*
☒	☐	A description of all covariates tested
☐	☒	A description of any assumptions or corrections, such as tests of normality and adjustment for multiple comparisons
☐	☒	A full description of the statistical parameters including central tendency (e.g. means) or other basic estimates (e.g. regression coefficient) AND variation (e.g. standard deviation) or associated estimates of uncertainty (e.g. confidence intervals)
☐	☒	For null hypothesis testing, the test statistic (e.g. *F*, *t*, *r*) with confidence intervals, effect sizes, degrees of freedom and *P* value noted<br>*Give P values as exact values whenever suitable.*
☒	☐	For Bayesian analysis, information on the choice of priors and Markov chain Monte Carlo settings
☒	☐	For hierarchical and complex designs, identification of the appropriate level for tests and full reporting of outcomes
☒	☐	Estimates of effect sizes (e.g. Cohen's *d*, Pearson's *r*), indicating how they were calculated

Our web collection on statistics for biologists contains articles on many of the points above.

Software and code

Policy information about availability of computer code

Data collection	No specific software was used for data collection.
Data analysis	Canu v1.7<br>Pilon v1.22<br>BLASTN v2.9.0<br>ALLHiC assembler v0.9.8<br>BWA-MEM v0.7.17<br>BUSCO v4<br>PASA v.r20140417<br>MUMmer v4.0.0-beta2<br>SnpEff v4.3t<br>XP-CLR v1.0<br>Assemblytics v1.2<br>ADMIXTURE v1.3.0<br>MOSAIC v1.3.8<br>Trimmomatic v0.38<br>HiCUP pipeline v0.8.2<br>Bowtie2 v2.3.5<br>RepeatMasker v4.1.0<br>SignalP v5.0<br>ApoplastP v1.0.1

Phobious v1.01
TargetP v2.0
TMHMM v2.0
HISAT2 v.2.2.1
GATK pipeline v4.1.4.1
BCFtools v.1.9
Plink v1.9
R package PopGenome v2.7.5
SNPgenie v2019.10.3
Jellyfish v2.2.10
JVarkit v.1.0
R package vcfR v1.12.0
R package adegenet v2.1.3
R package ape v5.4
R package ggtree v2.2.4
Beagle.28Jun21.220.jar
vcftools v0.1.13
SweeD v4.0.0
R package pophelper v2.3.0
samtools v1.8
guppy software v. 5.0.11
Dotplotly v.10.29.2017
SMC++ v1.15.4.dev17+g2ef0edb
MaCS v.0.4f
msstats v0.3.5

Custom scripts used in the study are deposited to GitHub: https://github.com/akhunovlab/stem_rust_diversity

For manuscripts utilizing custom algorithms or software that are central to the research but not yet described in published literature, software must be made available to editors and reviewers. We strongly encourage code deposition in a community repository (e.g. GitHub). See the Nature Portfolio guidelines for submitting code & software for further information.

Data

Policy information about availability of data

All manuscripts must include a data availability statement. This statement should provide the following information, where applicable:

- Accession codes, unique identifiers, or web links for publicly available datasets
- A description of any restrictions on data availability
- For clinical datasets or third party data, please ensure that the statement adheres to our policy

Data accessibility: The full genome assembly of Pgt isolate 99KS76A-1 is available at NCBI under accession number PGFY00000000.2 [https://www.ncbi.nlm.nih.gov/nuccore/PGFY00000000.2/] and GCA_002762355.2 [https://www.ncbi.nlm.nih.gov/assembly/GCA_002762355.2] associated with BioProject PRJNA313186 [https://www.ncbi.nlm.nih.gov/bioproject/PRJNA313186/]. Sequencing data used for genome assembly, including Oxford Nanopore long read data, PacBio data, Hi-C data and Miseq reads, have been deposited to NCBI under BioProject PRJNA313186. The Hi-C contact frequency data is available from NCBI GEO GSE210552 [https://www.ncbi.nlm.nih.gov/geo/query/acc.cgi?acc=GSE210552]. RNA-seq or CDS data for genome annotation were downloaded from NCBI BioProjects PRJNA415853 [https://www.ncbi.nlm.nih.gov/search/all/?term=PRJNA415853], PRJNA516922 [https://www.ncbi.nlm.nih.gov/search/all/?term=PRJNA516922], PRJNA66375 [https://www.ncbi.nlm.nih.gov/search/all/?term=PRJNA66375], and PRJNA347320 [https://www.ncbi.nlm.nih.gov/search/all/?term=PRJNA347320]. Illumina sequence files for the Pgt diversity panel deposited to NCBI under BioProject PRJNA803546 [https://dataview.ncbi.nlm.nih.gov/object/PRJNA803546].

Code availability: Custom scripts used in the study are deposited to GitHub: https://github.com/akhunovlab/stem_rust_diversity 94.

Human research participants

Policy information about studies involving human research participants and Sex and Gender in Research.

Reporting on sex and gender	*Use the terms sex (biological attribute) and gender (shaped by social and cultural circumstances) carefully in order to avoid confusing both terms. Indicate if findings apply to only one sex or gender; describe whether sex and gender were considered in study design whether sex and/or gender was determined based on self-reporting or assigned and methods used. Provide in the source data disaggregated sex and gender data where this information has been collected, and consent has been obtained for sharing of individual-level data; provide overall numbers in this Reporting Summary. Please state if this information has not been collected. Report sex- and gender-based analyses where performed, justify reasons for lack of sex- and gender-based analysis.*
Population characteristics	*Describe the covariate-relevant population characteristics of the human research participants (e.g. age, genotypic information, past and current diagnosis and treatment categories). If you filled out the behavioural & social sciences study design questions and have nothing to add here, write "See above."*
Recruitment	*Describe how participants were recruited. Outline any potential self-selection bias or other biases that may be present and how these are likely to impact results.*
Ethics oversight	*Identify the organization(s) that approved the study protocol.*

Note that full information on the approval of the study protocol must also be provided in the manuscript.

Field-specific reporting

Please select the one below that is the best fit for your research. If you are not sure, read the appropriate sections before making your selection.

☐ Life sciences ☐ Behavioural & social sciences ☒ Ecological, evolutionary & environmental sciences

For a reference copy of the document with all sections, see nature.com/documents/nr-reporting-summary-flat.pdf

Ecological, evolutionary & environmental sciences study design

All studies must disclose on these points even when the disclosure is negative.

Study description	Using haplotype-resolved genome assembly of a US isolate of Puccinia graminis f.sp. tritici (Pgt) as a reference, we characterized the structure variants (SVs) and single polymorphisms (SNPs) in a diverse panel of Pgt isolates with the purpose of investigating impacts of SVs and past admixture events on Pgt virulence.
Research sample	A panel of 77 geographically diverse Pgt isolates.
Sampling strategy	The sample includes Pgt isolates from the US and the other parts of the world and was selected to represent the major Pgt clades previously identified in genetic studies, as well as to represent a sexually recombining population derived from two diverged lineages of rust.
Data collection	The virulence of each of the Pgt isolates was assessed by infecting the panel of wheat differential lines carrying known stem rust resistance genes (Sr) by M. N. Rouse. The wheat seedlings were infected and scored at the USDA Cereal Disease Laboratory (CDL) at a biosafety level-3 facility (University of Minnesota). The Pgt urediniospores stored at -80C were heat-shocked for 6-10 min in a water bath at 42C, resuspended in Soltrol 170 light oil (Chevron Phillips Chemical Company, The Woodlands, TX). Inoculations were performed by air-spray at one-leaf stage. Immediately after inoculation, plants were transferred to a dew chamber and incubated in the dark for 16 h at 22C and under 100% relative humidity. The seeclings were transferred to greenhouse and grown at 22C day and 18C night with 16 h of photoperiod. The Pgt virulence scores ranging from 0 (highly avirulent) to 9 (highly virulent) on each differential were assessed 12-14 days after inoculation.
Timing and spatial scale	The Pgt isolates were taken from the collection assembled by the USDA ARS Cereal Disease Lab over last five decades. The collection includes isolates collected in the US as well as isolates collected in Europe, Africa, and Asia.
Data exclusions	Pgt isolates, 76W1142A and 83SYR2-2, were removed from majority of the analyses due to mislabeling, except for some population level statistics not affected by mislabeling, e.g LD decay calculation and global Pgt diversity analyses.
Reproducibility	The evaluation of disease resistance score in each experiment is based on at least 5 plants. For 31 out 79 isolates, these scoring experiments were replicated at least twice. Virulence scores collected across these replicated experiments were highly correlated ($r > 0.9$) indicating that the procedures used in the study generate reproducible data.
Randomization	Not applicable. All isolates were screened on the same panel of differential wheat lines carrying a set of known resistance genes. Thus, all possible combinations of Pgt isolates and wheat host genotypes was explored.
Blinding	During disease resistance scoring, the wheat host genotypes used for inoculation with Pgt isolates did not have labels visible to a researcher.

Did the study involve field work? ☐ Yes ☒ No

Reporting for specific materials, systems and methods

We require information from authors about some types of materials, experimental systems and methods used in many studies. Here, indicate whether each material, system or method listed is relevant to your study. If you are not sure if a list item applies to your research, read the appropriate section before selecting a response.

Materials & experimental systems

n/a	Involved in the study
☒	☐ Antibodies
☒	☐ Eukaryotic cell lines
☒	☐ Palaeontology and archaeology
☒	☐ Animals and other organisms
☒	☐ Clinical data
☒	☐ Dual use research of concern

Methods

n/a	Involved in the study
☒	☐ ChIP-seq
☒	☐ Flow cytometry
☒	☐ MRI-based neuroimaging
